# Rationale and Design of the First Double-Blind, Placebo-Controlled Trial with Allogeneic Adipose Tissue-Derived Stromal Cell Therapy in Patients with Ischemic Heart Failure: A Phase II Danish Multicentre Study

**DOI:** 10.1155/2017/8506370

**Published:** 2017-09-19

**Authors:** Jens Kastrup, Morten Schou, Ida Gustafsson, Olav W. Nielsen, Rasmus Møgelvang, Klaus F. Kofoed, Charlotte Kragelund, Jens Dahlgaard Hove, Andreas Fabricius-Bjerre, Merete Heitman, Mandana Haack-Sørensen, Lisbeth Drozd Lund, Ellen Mønsted Johansen, Abbas Ali Qayyum, Anders Bruun Mathiasen, Annette Ekblond

**Affiliations:** ^1^Department of Cardiology, The Heart Centre, Rigshospitalet, University of Copenhagen, Copenhagen, Denmark; ^2^Cardiology Stem Cell Centre, The Heart Centre, Rigshospitalet, University of Copenhagen, Copenhagen, Denmark; ^3^Department of Cardiology, Herlev Hospital, University of Copenhagen, Copenhagen, Denmark; ^4^Department of Radiology, Herlev Hospital, University of Copenhagen, Copenhagen, Denmark; ^5^Department of Cardiology, Hvidovre Hospital, University of Copenhagen, Copenhagen, Denmark; ^6^Department of Cardiology, Bispebjerg Hospital, University of Copenhagen, Copenhagen, Denmark; ^7^Department of Radiology, Rigshospitalet, University of Copenhagen, Copenhagen, Denmark; ^8^Department of Radiology, Hvidovre Hospital, University of Copenhagen, Copenhagen, Denmark; ^9^Department of Radiology, Bispebjerg Hospital, University of Copenhagen, Copenhagen, Denmark

## Abstract

**Background:**

Ischemic heart failure (IHF) has a poor prognosis in spite of optimal therapy. We have established a new allogeneic Cardiology Stem Cell Centre adipose-derived stromal cell (CSCC_ASC) product from healthy donors. It is produced without animal products, in closed bioreactor systems and cryopreserved as an off-the-shelf product ready to use.

**Study Design:**

A multicentre, double-blind, placebo-controlled phase II study with direct intramyocardial injections of allogeneic CSCC_ASC in patients with chronic IHF. A total of 81 patients will be randomised at 2 : 1 to CSCC_ASC or placebo. There is no HLA tissue type matching needed between the patients and the donors.

**Methods:**

The treatment will be delivered by direct injections into the myocardium. The primary endpoint is change in the left ventricle endsystolic volume at 6-month follow-up. Secondary endpoints are safety and changes in left ventricle ejection fraction, myocardial mass, stroke volume, and cardiac output. Other secondary endpoints are change in clinical symptoms, 6-minute walking test, and the quality of life after 6 and 12 months.

**Conclusion:**

The aim of the present study is to demonstrate safety and the regenerative efficacy of the allogeneic CSCC_ASC product from healthy donors in a double-blind, placebo-controlled, multicentre study in patients with IHF.

## 1. Background

Ischemic heart disease (IHD) caused by coronary artery disease is the most common cause of death with more than 17 million deaths worldwide each year and the major cause of hospital admissions [1]. It is an increasing economic health problem due to increasing morbidity in an ageing population. Conventional therapies have reduced the mortality of IHD significantly, but left an increasing number of patients with chronic IHD and/or ischemic heart failure (IHF) without further treatment options.

When looking for new treatment options, regenerative therapy with stem cells has been investigated intensively in several clinical studies in patients with acute or chronic IHD [2–4]. Several different cell populations have been investigated. Initially, it was mainly bone marrow-derived mononuclear cells (MNCs), mesenchymal stromal cells (MSCs), CD34^+^, CD133^+^, and endothelial progenitor cells. Later, cell populations have been isolated from the adipose tissue (adipose-derived mesenchymal stromal cells (ASCs)), placenta, umbilical cord, the heart, and several other tissues [2]. Presently, no consensus exists about the best cell type for clinical regenerative therapy.

The presence of MSC-like cells in all tissues of the body indicates their importance, and preclinical studies indicate that these cells have regenerative capacity regardless of tissue origin [4]. Most clinical studies have used BM-MSCs. However, from a feasibility point of view, ASCs are superior to BM-MSCs as a higher yield of ASCs can be isolated from abdominal adipose tissue compared to MSCs from the bone marrow. [5] Furthermore, ASCs grow faster than BM-MSCs during culture expansion [6].

We have conducted three clinical studies with autologous culture-expanded BM-MSCs and ASCs in patients with IHD with or without heart failure [7–11]. The treatments have been safe and the regenerative efficacy promising. The studies demonstrated improvements in left ventricular ejection fraction, heart tissue remodeling, exercise capacity, and relief of symptoms.

To implement and disseminate a new clinical therapy with stem cells to all potential candidates, safety and efficacy have to go hand in hand with feasibility; it has to be logistically easy to request and perform the treatment. However, there are many logistical obstacles in the presently used clinical models for autologous MSC therapies within cardiology: Invasive harvesting of bone marrow or aspiration of abdominal adipose tissue in all patients was followed by several weeks of culture expansion with a huge variation in proliferation rate and cell yield from patient to patient. This makes it difficult to treat patients with a standardised number of cells in clinical studies. Moreover, it is logistically difficult to schedule the treatment procedure due to large variations in cell production time.

Additionally, the number of cells delivered to the patient may be of importance for the treatment efficacy and the patients may need more than one treatment session to get relief from cardiac symptoms.

We have tackled these serious hurdles by establishing new ASC cultivation protocols with human platelet lysate in closed bioreactor systems and by establishing a centralised production of an allogeneic Cardiology Stem Cell Centre adipose-derived stromal cell (CSCC_ASC) product from healthy donors, which can be stored in nitrogen vapour containers as an off-the-shelf product in the hospital ready to be used without any delay.

This newly developed cryopreserved product, CSCC_ASC, from healthy donors was safe and feasible in a phase I study in patients with IHF [12]. In addition, we observed a tendency towards efficacy in patients although the study was not powered for that.

This study was established to investigate both the safety and the efficacy with regard to the regenerative potential of this newly developed CSCC_ASC product. The present report will outline the rationale and design of this multicentre, double-blinded, placebo-controlled phase II study using intramyocardial delivered allogeneic CSCC_ASC in patients with IHF.

## 2. Methods

### 2.1. Study Design and Population

The study protocol complies with the Declaration of Helsinki and was approved by the Danish National Committee on Health Research Ethics (number 1717872) and Danish Medicines Agency (EudratCT number 2015-001560-19). The study is registered with clinicalTrials.gov (NCT02387723). The local Good Clinical Practice Unit from the capital region of Denmark will monitor the study.

The study will include 81 patients with IHF between 30 and 85 years of age with reduced left ventricular ejection fraction (LVEF) (≤45%) evaluated by echocardiography, New York Heart Association (NYHA) classes II-III, no further revascularization options and on maximal tolerable heart failure medication. Inclusion and exclusion criteria are described more in detail in Table 1.

The patients will be screened and followed locally in the four participating clinical cardiology sites. All patients will be treated centrally at the Department of Cardiology, Rigshospitalet University Hospital Copenhagen in a 2 : 1 randomisation with either CSCC_ASC or placebo (isotonic saline).

**Figure 1:**
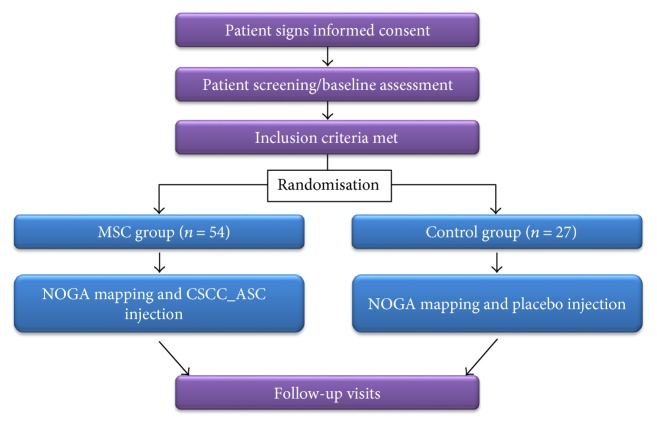
Design of the MSC-HF II trial. CSCC_ASC: cardiology stem cell centre adipose-derived stromal cells.

Patients will have follow-up visits 1, 3, 6, and 12 months after treatment for safety and efficacy evaluation (Figure 1 and Table 2).

### 2.2. Cell Production

The investigational cell product, CSCC_ASC, will be produced in the Cardiology Stem Cell Centre, Rigshospitalet University Hospital Copenhagen in an approved good manufacturing practice (GMP) facility following the description in an approved Investigational Medicinal Product Dossier.

The cell product will derive from 5 to 7 healthy donors. The donor eligibility will be determined by a donor interview, a questionnaire, and testing for infectious disease markers including human immunodeficiency virus (HIV) and human T-cell lymphotropic virus (HTLV) types I/II by serum analyses within 30 days prior to liposuction. In addition, a blood sample will be drawn on the day of donation for repeated serology and nucleic acid testing of HIV and hepatitis B and C.

The donors will have an abdominal liposuction (100–150 mL of lipoaspirate). The preparation of lipoaspirates, isolation of stromal vascular fractions, and expansion of adipose-derived stromal cells in Quantum Cell Expansion Systems (Terumo, US) will be performed as previously described [12–14]. Each CSCC_ASC treatment vial will consist of ASCs from one donor only.

The production unit will produce and deliver both the CSCC_ASC and the placebo (isotonic saline) batches based on the randomisation code. It will label the Investigational Medicinal Product (IMP) and placebo in accordance with the legislation and keep the randomisation code until finalization of the clinical trial. The final cell products will be stored in nitrogen vapour containers until clinical use.

### 2.3. Cell Treatment

There will be no humane leucocyte antigen (HLA) tissue type matching between the donor and the patients. The IMP will be thawed and prepared for injection immediately before treatment. The treatment ampoules are transparent, and the colour of the placebo product is slightly different from that of the cell product. Therefore, to assure the blinding of the treatment, the clinical teams which are responsible for the screening and follow-up of the patients will not participate in the treatment procedure or be in the catheterization laboratory during the treatment. The staff in the catheterization laboratory will be instructed to avoid comments on the delivered product.

### 2.4. NOGA-Guided Injection

A 3D map of the left ventricle will be created using the NOGA XP® system (Biological Delivery System, Cordis, Johnson & Johnson, USA) [7–10]. With the MYOSTAR® injection catheter (Biological Delivery System, Cordis, Johnson & Johnson, USA), the IMP (100 million CSCC_ASCs or placebo) will be delivered into the myocardium by 12–20 injections of 0.3–0.4 mL. The injections will be performed in the viable myocardial tissue with a unipolar voltage > 6 mV. Plasma CKMB and Troponin will be measured before, immediately after, 6 hours and at day 1 after the injections in order to assess possible myocardial effects.

### 2.5. ECHO and CT Scans

Patients will undergo cardiac computed tomography (CT) and echocardiography (ECHO) scans with contrast at baseline and six months after treatment [8]. For cardiac CT (320 slice, Aquilion, Toshiba, Tokyo, Japan, or Flash dual source, Siemens, Siemens Healthcare GmbH, Germany), the R-R interval and multisegmental image reconstruction will be performed with the most recent scanner software. ECHO will use parasternal and apical two 2D and 3D views. CT scans with contrast will not be performed if plasma creatinine > 130 *μ*mol/L or in patients with known contrast allergy.

All image data will be stored in a central server and be analysed blinded. CT scans will be analysed with the cvi42 postprocessing tool (Circle Cardiovascular Imaging, Calgary, Alberta, Canada). Endocardial and epicardial borders will be traced manually in end-diastole and end-systole starting from the mitral plane and ending at apex. ECHO will be analysed using IntelliSpace Cardiovascular (Philips Medical Systems, Best, the Netherlands) to obtain conventional as well as deformation and 3D volumetric measures.

### 2.6. Biomarkers and Alloantibodies

All donors will have an intermediate resolution typing of human leucocyte antigen- (HLA-) A, HLA-B, HLA-C, DRB1, DRB345, DQA1, DQB1, DPA1, and DPB1 loci by real-time PCR with subsequent melting point analyses using a Linkseq 384-well complete typing kit (Linkage Biosciences Inc., CA, US). Patient blood samples are drawn at baseline and follow-up and will be stored for later centralized analyses of biomarkers and the presence of alloantibodies with the LABScreen HLA classes I and II single antigen bead assay on a Luminex 100 (One Lambda Inc., Thermo Fischer, Canoga/Los Angeles, CA, USA).

### 2.7. Endpoints

The *primary endpoint* is the difference between the two groups of the change in left ventricle end-systolic volume (LVESV) from baseline to 6-month follow-up measured with CT scans and with ECHO scans in patients with reduced kidney function.

The *secondary endpoints* are safely evaluated by the development of alloantibodies and laboratory safety measurements, incidence and severity of serious adverse events, and suspected unrelated serious adverse events 1, 3, 6, and 12 months after treatment. Other endpoints are changes in LVEF, left ventricle end-diastolic volume (LVEDV), stroke volume, cardiac output, and end-diastolic myocardial mass at 6-month follow-up. The changes in left ventricle function will be measured by CT and ECHO.

Other secondary endpoints are changes in NYHA and CCS class, Kansas City Cardiomyopathy Questionnaire (KCCQ), Seattle Angina Questionnaire, EQ5D3L Questionnaire, 6-minute walking test, and NT-pro-BNP.

We will also asses changes in the following endpoints alone and combined:

(1) Death, hospitalization for worsening heart failure including implanting of a biventricular pacemaker, and hospitalization because of ventricular tachycardia or fibrillation 1, 2, and 3 years after treatment.

(2) Death, hospitalization for any cardiovascular reason, hospitalization for worsening heart failure including inserting of a biventricular pacemaker, and hospitalization because of ventricular tachycardia or fibrillation 1, 2, and 3 years after treatment.

### 2.8. Power Calculation

#### 2.8.1. Definition and Justification (Power Calculation) of Sample Size

The enrolment of 81 patients in a 2 : 1 randomisation (54 : 27) will have a statistical power above 90% for the detection of an absolute change between groups in LVESV of 10 mL expected SD of 13 mL and 11.6 mL with expected SD of 15 mL. For all, alpha values of 5% were used. Change between groups in LVEF of 3.1% with expected SD of 4% and 3.9% with expected SD of 5% will also have statistical power above 90% with a 5% alpha value.

Power calculations are based on our recently finalized double-blind, placebo-controlled trial with 60 patients with IHF (MSC-HF trial); we compared direct intramyocardial injections of autologous MSCs or placebo in a 2 : 1 randomisation design. The six-month follow-up demonstrated significant improvement in LVESV with a difference between groups of 13.0 ± 12.9 mL (*p* = 0.001). There were also significant improvements in LVEF with a difference of 6.2 ± 3.8% (*p* < 0.0001) between MSC and placebo-treated patients.

In the MSC-HF trial, cardiac measurements were done by magnetic resonance imaging (MRI) and CT. Since the SD is often a little higher for echocardiographic measured parameters compared to the data obtained using MRI and CT, we used slightly higher SD in our power calculations.

#### 2.8.2. Safety and Ethical Considerations

The Cardiology Stem Cell Centre has conducted several clinical studies with direct injection of stem cells or genes into the myocardium with the methodology employed without any safety issues raised [7–10].

The newly developed CSCC_ASC product has been evaluated in 10 patients with IHF without any safety concerns [12]. Each patient was treated with cells from one of three donors, and no matching between the donor and the patient tissue types was performed. There were no procedure-related complications to the direct intramyocardial injection of CSCC_ASC. During the 6-month follow-up period, four out of ten patients developed donor-specific de novo HLA class I antibodies and two other patients had prior to the treatment donor-specific antibodies. None of the patients had any clinical symptoms, changes in biochemical parameters, or inflammatory signs indicating an immunization.

Based on these data and the safety data from other groups using allogeneic MSC therapy in patients, we consider it safe to continue with the present clinical phase II trial.

During the two CT scans, patients may receive a radiation dose of up to 20 mSv depending on body weight, heart rate, and regularity of the heart rate during the scan. It can be calculated that the lifetime risk of dying from cancer hereby theoretically increases by 0.1%. The patients receiving a maximum radiation dose of 20 mSv will have their lifetime risk of dying from cancer increased from 25% to 25.1%. The above estimation is based on calculations done by the National Institute of Radiation Protection, Denmark.

## 3. Discussion

The present described study is the first double-blinded, randomised, multicentre, placebo-controlled trial to test the efficacy and safety of a newly developed intramyocardial delivered allogeneic adipose-derived mesenchymal stromal cell product CSCC_ASC from healthy donors in patients with known chronic IHF. The primary efficacy endpoint is the difference between the two groups of the change in left ventricle end-systolic volume (LVESV) from baseline to 6-month follow up. The LVESV has been chosen based on the previous demonstration that treatment with beta-blockers can improve LVESV and that it is a strong prognostic parameter of death in patients with heart failure [15, 16].

The use of autologous MSCs for clinical therapy has been proven to be safe and with some efficacy as a regenerative treatment for ischemic heart patients in several clinical studies (Tables 3 and 4). However, based on collected experience from clinical trials, the lack of a homogenous cell product and dose, as well as timing and logistics around transportation of autologous cell therapy, prevents feasibility, repeatability, and dissemination of regenerative therapy. Therefore, a switch from autologous to allogeneic ASC therapy is necessary to propagate cell therapy clinically.

It is well known that there are some differences between BM-MSCs and ASCs, in their gene expression profile, their angiogenic potential, and secretion of factors [29]. It has been demonstrated that the most discriminative feature for heterogeneity within MSC cultures is the tissue source followed by the culture methodology [30, 31]. In spite of these differences, both cell sources seem to be equally effective clinically (Tables 3 and 4). Since ASCs can be obtained in substantially greater amounts, they may appear to be a better choice than BM-MSCs for clinical application [5, 6].

However, there are also many similarities between MSCs from various tissue sources in morphology, kinetics, surface markers, multipotency, and most intriguingly immune modulatory capacities [5, 32–35]. Cells are believed to be able to evade being recognized by the recipient immune system via modulating the recipient immune function. This feature makes MSCs worthy candidates for allogeneic therapy [33–35]. MSCs engage in direct cell-cell interaction with antigen-presenting cells and secrete a number of factors to modulate the local environment in a trophic manner. Allogeneic MSCs and ASCs have already been used in clinical trials without any side effects (Tables 3 and 4). Un-blinded comparative studies between autologous and allogeneic BM-MSCs in patients with heart failure have demonstrated safety and clinically meaningful efficacy of allogeneic BM-MSC versus autologous BM-MSC [22, 24].

Moreover, culture expansion of MSCs is presently performed in open flasks of varying sizes. The manual procedures needed for changing media are time consuming and therefore labour extensive, and all pose a great risk for contamination. Consequently, implementing newly available closed bioreactor systems with continuous delivery of medium will minimise contamination risk and improve the standardisation of the final clinical cell product.

At Cardiology Stem Cell Centre, Rigshospitalet University Hospital Copenhagen, we have established new cultivation protocols with human platelet lysate in closed bioreactor systems for the production of allogeneic CSCC_ASC from healthy donors, which can be cryopreserved and stored in nitrogen vapour containers as an off-the-shelf product ready to be used.

This newly developed product has a first in man safety study in patients with IHF-demonstrated safety, feasibility, and a tendency towards efficacy [12]. Based on these data and the knowledge from other clinical studies using allogeneic mesenchymal cell products for treatment, we have found it relevant to test the safety and efficacy of CSCC_ASC in a larger placebo-controlled trial in patients with IHF. If the same safety and efficacy profile of the present allogeneic mesenchymal cell product stored as an off-the-shelf product as seen with autologous MSCs can be demonstrated, then it will be an important step towards the implementation of cell therapy in severely sick IHF patients.

## Figures and Tables

**Table 1:** Inclusion and exclusion criteria.

*Inclusion criteria*
(1) 30 to 85 years of age
(2) Signed informed consent
(3) Chronic stable ischemic heart disease
(4) Symptomatic heart failure (NYHA II-III)
(5) Left ventricle ejection fraction (LVEF) ≤ 45% documented by echocardiography at randomisation obtained after up-titration in heart failure medication (if cardiac resynchronisation therapy device (CRT) 3 months after implantation)
(6) Plasma NT-pro-BNP > 300 pg/mL (>35 pmol/L) in sinus rhythm and plasma NT-pro-BNP > 422 pg/mL (>49 pmol/L) in patients with atrial fibrillation
(7) Maximal tolerable heart failure medication
(8) Heart failure medication unchanged two months prior to inclusion/signature of informed consent. Changes in diuretics accepted
(9) No option for percutaneous coronary intervention (PCI) or coronary artery bypass graft (CABG)
(10) Patients who have had PCI or CABG within six months of inclusion must have a new angiography less than one month before inclusion and at least four months after the intervention to rule out early restenosis
(11) Patients cannot be included until three months after implantation of a CRT or 1 month after an implantable cardioverter device (ICD)
*Exclusion criteria*
(1) Heart failure (NYHA I or IV)
(2) Acute coronary syndrome with elevation of CKMB or troponins, stroke, or transitory cerebral ischemia within six weeks of inclusion
(3) Other revascularization treatment within four months of treatment
(4) If clinically indicated that the patient should have a coronary angiography before inclusion
(5) Moderate to severe aortic stenosis (valve area < 1.3 cm^2^) or valvular disease with option for surgery
(6) Diminished functional capacity for other reasons such as obstructive pulmonary disease with forced expiratory volume in 1 second (FEV1) < 1 L/min, moderate to severe claudication, severe arthrosis or severe pain from the musculoskeletal system, or morbid obesity
(7) Clinical significant anaemia (haemoglobin < 6 mmol/L), leukopenia (leucocytes < 2 × 10^9^/L), leucocytosis (leucocytes > 14 × 10^9^/L), or thrombocytopenia (thrombocytes < 50 × 10^9^/L)
(8) Anticoagulation treatment that cannot be paused during cell injections
(9) Patients with reduced immune response
(10) History with malignant disease within five years of inclusion or suspected malignancy, except treated skin cancer other than melanoma
(11) Pregnancy or lactation
(12) Other experimental treatment within four weeks of baseline tests
(13) Participation in another intervention trial
(14) Known hypersensitivity to dimethyl sulfoxide (DMSO)

**Table 2:** MSC-HF II study: summary of events.

Event	Screening/baseline	D1	D2	D3	M1	M3	M6	M12
Informed consent	X							
History/physical	X	X		X	X	X	X	X
NYHA	X	X			X	X	X	X
CCS	X	X			X	X	X	X
ECG	X	X		X	X	X	X	X
Biochemistry local laboratory	X	X	X	X	X	X	X	X
Biomarkers	X	X			X	X	X	X
HLA tissue typing	X							
HLA tissue antibodies	X				X	X	X	X
6-minute walking test	X					X	X	X
Chest X-ray	X							
CCT	X						X	X
KCCQ	X					X	X	X
SAQ	X					X	X	X
EQ5D3L	X					X	X	X
NOGA mapping			X					
IMP injection			X					
Echocardiography	X		X				X	
Holter monitoring	X	X	X	X				
AE evaluation			X	X	X	X	X	X

D: day; M: month; AE: adverse events; CCS: Canadian Cardiovascular Society functional classification; CCT: cardiac computed tomography imaging; ECG: electrocardiogram; HLA: humane leucocyte antigen; IMP: investigational medicinal product; KCCQ: Kansas City Cardiomyopathy Questionnaire; NYHA: New York Heart Association functional classification; SAQ: Seattle Angina Questionnaire. EQ5D3L questionnaire.

**Table 3:** Mesenchymal stromal cell trials in patients with acute myocardial infarction.

Reference	Number of patients	Cell type	Design	Delivery route	Endpoints
*Allogeneic*					
Hare et al. 2009 [17]	53	MSC	RPCT	IV	Safe. Improved FEV1
*Autologous*					
Chen et al. 2004 [18]	69	MSC	RPCT	IC	Improved LVEF, LVESV, perfusion
Katritsis et al. 2007 [19]	22	MSC	Open	IC	Improved wall motion and perfusion
Yang et al. 2010 [20]	16	MSC	Open	IC	Safe and feasible. No clinical improvement
Houtgraaf et al. 2012 [21]	14	MSC	RPCT	IC	Improved perfusion and reduced scar tissue

FEV1: forced expiratory volume in 1 second; IC: intracoronary; IV: intravenous; LV: left ventricular; LVEF: left ventricular ejection fraction; RPCT: randomised placebo controlled trial.

**Table 4:** Mesenchymal stem cell trials in patients with chronic ischemic heart disease.

Reference	Number of patients	Cell type	Design	Delivery route	Endpoints
*Allogeneic*					
Hare et al. 2012 [22]	30	MSC	RO	IM	Safety and feasible
Perin et al. 2015 [23]	60	MSC	RSCT	IM	Safety and feasible
Kastrup et al. 2017 [12]	10	ASC	Open	IM	Safety and feasible
Hare et al. 2017 [24]	37	MSC	Open	IM	Safety and efficacy
*Autologous*					
Chen et al. 2006 [25]	22	MSC	Open	IC	Improved LVEF, exercise capacity, and symptoms
Mohyeddin-Bonab et al. 2007 [26]	8	MSC	Open	IC	Improved LVEF, infarct size, and symptoms
Katritsis et al. 2007 [19]	5	MSC	Open	IC	Not arrhythmogenic
Williams et al. 2011 [27]	4	MSC	Open	IM	Reduced LV dimensions and infarct size
Friis et al. 2011 [7]	31	MSC	Open	IM	Improved LVEF, exercise capacity, and symptoms
Lasala et al. 2011 [28]	10	MSC	Open	IC	Increased LVEF
Mathiasen et al. 2014 [10]	60	MSC	PPCT	IM	Safe, improved LVEF, and increased myocardial mass
Qayyum et al. 2017 [11]	60	ASC	PPCT	IM	Safe. Tendency toward improved exercise capacity

IC: intracoronary; IM: intramyocardial; LV: left ventricular; LVEF: left ventricular ejection fraction; RO: randomised open study; RPCT: randomised placebo controlled trial; RSCT: randomised sham controlled trial.
